# Clinical outcomes of hypofractionated image-guided multifocal irradiation using volumetric-modulated arc therapy for brain metastases

**DOI:** 10.1093/jrr/rry091

**Published:** 2018-11-17

**Authors:** Shunsuke Furutani, Hitoshi Ikushima, Motoharu Sasaki, Chisato Tonoiso, Ayaka Takahashi, Akiko Kubo, Takashi Kawanaka, Masafumi Harada

**Affiliations:** 1 Department of Radiology, Institute of Biomedical Sciences, Tokushima University Graduate School, 3-18-15 Kuramoto-cho, Tokushima, Japan; 2 Department of Radiation Therapy Technology, Institute of Biomedical Sciences, Tokushima University Graduate School, 3-18-15 Kuramoto-cho, Tokushima, Japan; 3 Department of Radiological Technology, Tokushima University Hospital, 3-18-15 Kuramoto-cho, Tokushima, Japan

**Keywords:** hypofractionated radiotherapy, volumetric-modulated arc therapy, brain metastases, linear accelerator

## Abstract

Volumetric-modulated arc therapy (VMAT) can be used to design hypofractionated radiotherapy treatment plans for multiple brain metastases. The purpose of this study was to evaluate treatment outcomes of hypofractionated image-guided multifocal irradiation using VMAT (HFIGMI–VMAT) for brain metastases. From July 2012 to December 2016, 67 consecutive patients with 601 brain metastases were treated with HFIGMI–VMAT at our institution. The prescribed dose was 50 Gy to a 95% volume of the planning target volume in 10 fractions. Fifty-five of the 67 patients had non-small-cell lung cancer, and the remaining 12 had other types of cancer. The median number of brain metastases was five, and the median maximum diameter was 1.2 cm. The median duration of follow-up was 12.0 months (range, 1.9–44.8 months), and the median survival time 18.7 months. Four patients with six lesions had local recurrences. The local control rate in the 64 assessed patients was 98.4% and 95.3% at 6 and 12 months, respectively (three died before assessment). The local control rate for the 572 assessed lesions was 99.8% and 99.3% at 6 and 12 months, respectively. Thirty-nine patients developed distant brain metastases, the distant brain control rate being 59.7% and 40.5% at 6 and 12 months, respectively. Acute toxicities were generally mild (Grade 1–2). Three patients (4.5%) developed radiation necrosis requiring corticosteroid therapy. The HFIGMI–VMAT technique with flat dose delivery was well tolerated and achieved excellent local control. This technique is a promising treatment option for patients with multiple and large brain metastases.

## INTRODUCTION

Brain metastases occur in 20–40% of patients with cancer [1], their incidence having been increasing with developments in neuroimaging techniques and advances in systemic treatment regimens that have improved survival and control of extracranial metastatic disease [2–4]. As systemic control has improved, control of brain metastases has become more important. The prognosis of patients with brain metastases is poor; the median duration of survival being 3–4 months [5].

The main treatment options for brain metastases are surgery, stereotactic radiosurgery (SRS) and whole-brain radiotherapy (WBRT) [6]. Although WBRT has traditionally been the mainstay of treatment for multiple brain metastases, the dose administered by WBRT is insufficient to achieve long-term tumor control, and the subsequent prognosis is poor, median survival ranging from 3 to 7 months [7, 8]. SRS is a well-established means of treating brain metastases. However, the indications for SRS are generally limited by the number of lesions and tumor volumes [9, 10]. Volumetric-modulated arc therapy (VMAT) improves dose conformity and decreases dose to the surrounding healthy tissue, allowing escalation of the dose to the target volume [11, 12]. VMAT can deliver high-dose radiation to multiple targets simultaneously and can thus be used to design treatment plans for multiple brain metastases in a single-fraction or fractionated manner [13].

Most published studies have investigated the use of VMAT as WBRT with simultaneous integrated boost (SIB) or WBRT followed by VMAT boost to metastatic lesions [14–17]. Several groups have reported planning studies or clinical experience of VMAT as single-fraction radiosurgery [18–20]; however, there are few published studies that have addressed the clinical experience of VMAT as hypofractionated radiotherapy [21]. In the present study, we evaluated the efficacy and toxicity of hypofractionated image-guided multifocal irradiation using VMAT (HFIGMI–VMAT) with flat dose delivery and 3 mm planning target volume (PTV) margin in patients with brain metastases.

## MATERIALS AND METHODS

### Patients

This retrospective study included 67 consecutive patients with brain metastases (601 lesions) treated with HFIGMI–VMAT at our institution between July 2012 and December 2016. The eligibility criteria were histological diagnosis of a primary solid tumor, single or multiple brain metastases with a diameter of ≤3 cm, and total tumor volume of ≤15 cm^3^, with no limit on the number of lesions. Basically, in our institution HFIGMI–VMAT is recommended for the treatment of brain metastases when there are four or more, and/or the metastases are >1 cm in diameter. Patients who had had previous radiation therapy were eligible, whereas those with small-cell lung cancer or leptomeningeal metastases were excluded from this study. Fifty-five of the 67 patients had non–small-cell lung cancer and the remaining twelve other types of cancer. The median number of brain metastases per patient was five, and their median maximum diameter was 1.2 cm. Twenty-four patients had previously received cranial radiation treatment, nine of them having received WBRT. The patient characteristics are summarized in Table 1. This retrospective study was approved by the Institutional Review Board of our institution (Approval No. 2401).

**Table 1.** Patient characteristics

Characteristics	Data
Sex (*n*)	
Male	36
Female	31
Age (years)	
Median	68
Range	12–88
KPS score (*n*)	
<70	15
≥70	52
Extracranial metastases (*n*)	
Absent	23
Present	44
Primary tumor (*n*)	
Lung	55
Breast	5
Other	7
Number of BM (*n*)	
Median	5
Range	1–73
Maximum diameter of BM (cm)	
Median	1.2
Range	0.4–3.0
Number and maximum diameter of BM (*n*)	
≥4 and >1 cm	29
≥4 and ≤1 cm	18
<4 and >1 cm	16
<4 and ≤1 cm	4
Total volume of BM (cm^3^)	
Median	1.0
Range	0.1–14.1
Previous radiotherapy (*n*)	
WBRT	6
SRS	15
WBRT+SRS	3
No previous radiotherapy	43
Targeted therapy (*n*)	
No	37
Yes	30

KPS = Karnofsky performance status, BM = brain metastases, WBRT = whole brain radiation therapy, SRS = stereotactic radiosurgery.

### Treatment

All patients’ heads were immobilized with non-invasive thermoplastic head masks before they underwent treatment with Novalis Tx (Brainlab AG, Feldkirchen, Germany) or TrueBeam (Varian Medical Systems, Palo Alto, CA, USA) with 6 MV X-rays. The dose rate for these treatments was 600 MU/min. An ExacTrac patient positioning system (Brainlab AG) was used as a first step in patient positioning verification and correction with a 6D robotic couch, after which cone-beam computed tomography (CBCT) was performed for the first 3 days prior to treatment to reconfirm the target position. CBCT was subsequently omitted if corrections on the CBCT were not needed in the first 3 days. Diagnostic gadolinium-enhanced T1-weighted MRI (1.4-mm slice thickness) and planning CT images (2.5-mm slice thickness) were fused to delineate the targets and organ structure on iPlan software (Brainlab AG). The gross tumor volume (GTV) was defined as the contrast-enhancing volume on CT and MRI images; the clinical target volume being identical to the GTV. The PTV was generated by adding a 3-mm margin to the GTV. The contoured target and organ structures were transferred to an Eclipse treatment-planning system (Varian Medical Systems) for VMAT planning (RapidArc; Varian Medical Systems). Single isocenter two-axial coplanar arcs of 360° were used for VMAT treatment. The treatment beam-on time was ~3 min or less in all cases.

The prescribed dose was 50 Gy to a 95% volume of the PTV (D95) in 10 fractions with flat dose delivery (median D_2%_ is 108.1%). The dose fractionation schedule was based on previously reported doses of SRS, WBRT combined with SRS, and WBRT with SIB for multiple brain metastases [10, 22, 23]. The biologically effective dose (BED, α/β = 10 Gy) of 50 Gy in 10 fractions is close to 23 Gy in one fraction SRS, 30 Gy in 10 fractions WBRT combined with 15 Gy in one fraction SRS boost, or 50 Gy in 10 fractions SIB. The prescribed dose was reduced by 10–20% for tumors located in the brain stem and in patients who had previously received WBRT. The dose constraints to the organs at risk were as follows: brain stem <40 Gy, optic nerves and optic chiasm <35 Gy, eyes <30 Gy, and lens <10 Gy. The dosimetric results are summarized in Table 2.

**Table 2.** Details of dosimetry

	Min	Max	Median
PTV (% of prescription dose)			
D_2%_	104.4	135.2	108.1
D_98%_	86.0	99.3	98.8
D_50%_	101.7	128.0	104.5
GTV (% of prescription dose)			
D_50%_	101.8	133.8	105.9
Normal brain (Gy)			
D_mean_	1.0	32.9	10.4
Brain stem (Gy)			
D_2%_	0.1	45.0	20.2
D_mean_	0.0	28.7	8.7
Optic nerve (Gy)			
D_2%_	0.1	33.6	7.2
D_mean_	0.1	25.1	4.8
Optic chiasm (Gy)			
D_2%_	0.1	34.7	9.4
D_mean_	0.1	31.1	6.5
Eye (Gy)			
D_2%_	0.0	23.2	6.0
D_mean_	0.0	12.4	3.2
Lens (Gy)			
D_2%_	0.0	10.2	3.6
D_mean_	0.0	9.2	2.6

D_2%_ = dose to 2% of the volume, D_98%_ = dose to 98% of the volume, D_50%_ = dose to 50% of the volume, D_mean_ = mean dose of the volume.

### Follow-up and statistical methods

After treatment, all patients were followed-up with 2–3-monthly contrast-enhanced MRI scans and physician evaluation. Local recurrence was defined as a ≥20% enlargement in the size of treated lesions on MRI. Distant failure was defined as identification of new brain metastases. Neurological death was defined as progression of brain metastases or uncertain cause of death. Treatment-related toxicity was evaluated according to the National Cancer Institute Common Terminology Criteria for Adverse Events (CTCAE) v4.0 grading system. Local control and survival from the last date of radiotherapy were calculated using the Kaplan–Meier method. Comparisons of subgroups were performed using the log-rank test for univariate analysis and the Cox proportional hazard model for multivariate analysis. The following factors were analyzed for prognostic significance in local control rate, distant brain control rate, and overall survival: sex (male vs female), age (<65 years vs ≥65 years), Karnofsky Performance Status score (<70 vs ≥70), extracranial metastases (absent vs present), primary tumor site (lung vs others), number of brain lesions (≤four vs >four), total tumor volume (≤2 cm^3^ vs >2 cm^3^), previous WBRT (yes vs no), and targeted therapy (yes vs no). Statistical analysis was performed using EZR (Saitama Medical Center, Jichi Medical University, Saitama, Japan) [24], and *P* < 0.05 was regarded as denoting statistical significance.

## RESULTS

### Local control and survival

**Fig. 1.**
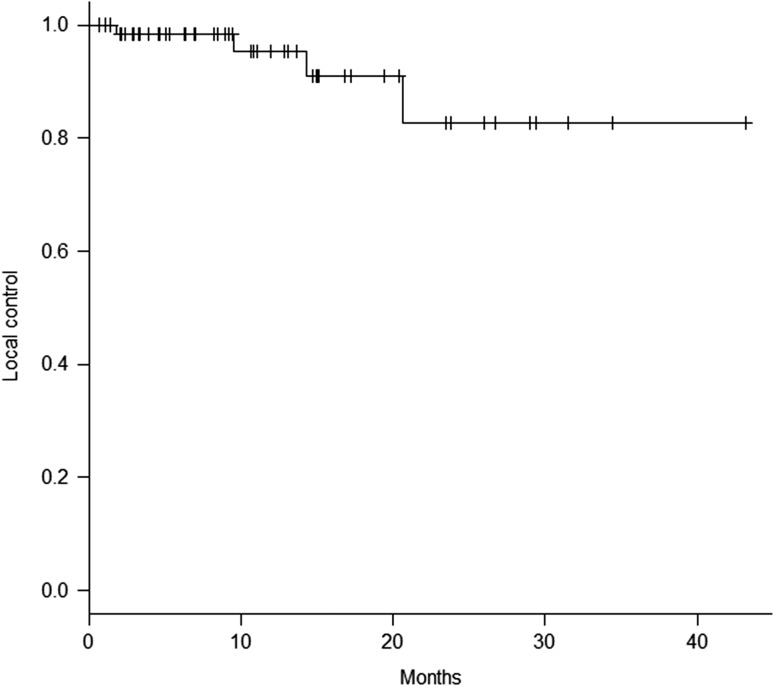
Local control achieved in the 64 assessed patients.

**Fig. 2.**
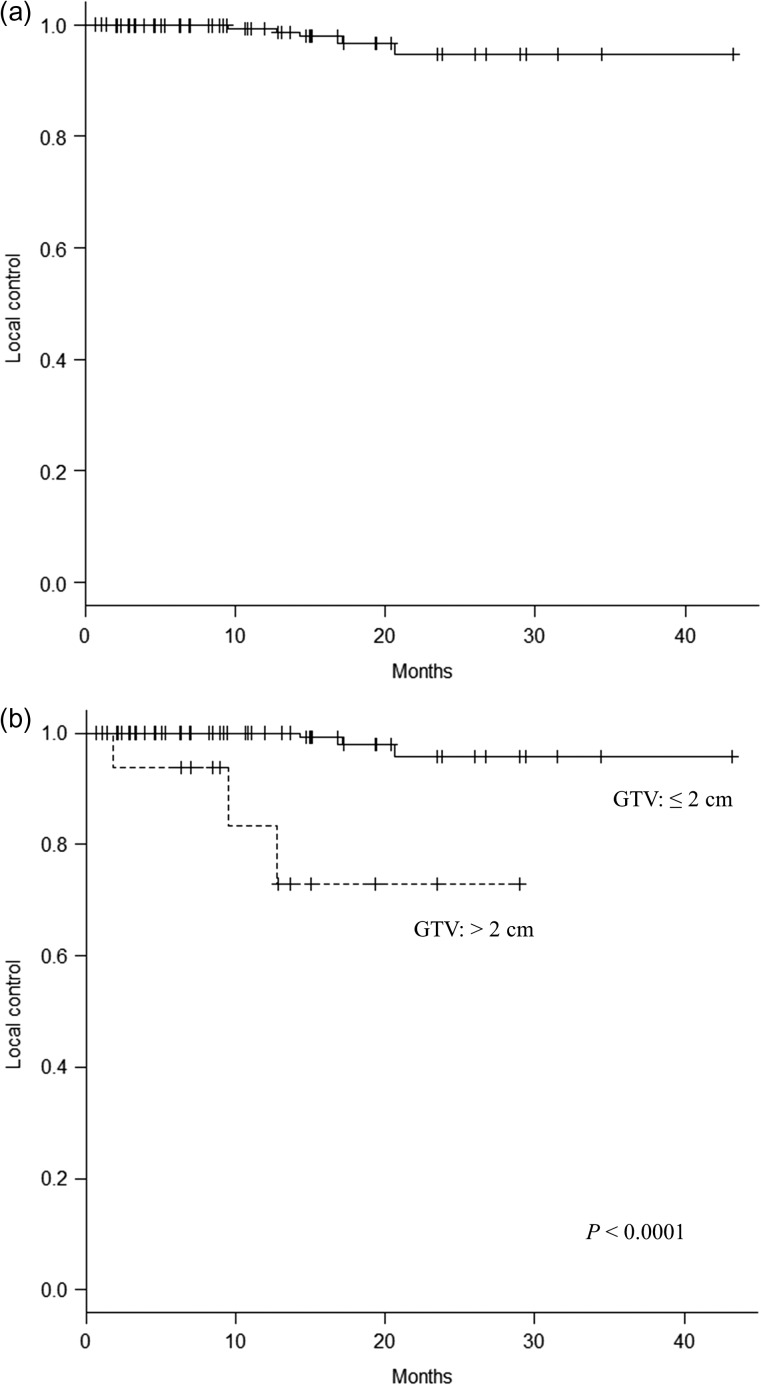
(a) Local control of 572 treated lesions. (b) Local control according to tumor diameter.

The median duration of follow-up was 12.0 months (range, 1.9–44.8 months). Three patients with a total of 29 lesions died before the first follow-up MRI scan and were therefore excluded from the local and distant control analysis. The median duration of imaging follow-up was 10.7 months (range, 0.6–43.2 months). Four patients developed local recurrences. The local control rate for the 64 assessed patients was 98.4% and 95.3% at 6 and 12 months, respectively (Fig. 1). According to univariate analysis, lung cancer, no previous WBRT, and total tumor volume of ≤2 cm^3^ were associated with better local control (*P* < 0.05). According to multivariate analysis, there was no significant association in any subset. Six lesions recurred locally; the local control rate for the 572 assessed lesions was 99.8% and 99.3% at 6 and 12 months, respectively (Fig. 2a). Figure 2b shows the local control rate according to tumor diameter. The local control rates at 12 months were 100% for tumors of diameter ≤2 cm and 83.3% for those of diameter >2 cm; this difference is statistically significant (*P* < 0.0001).

**Fig. 3.**
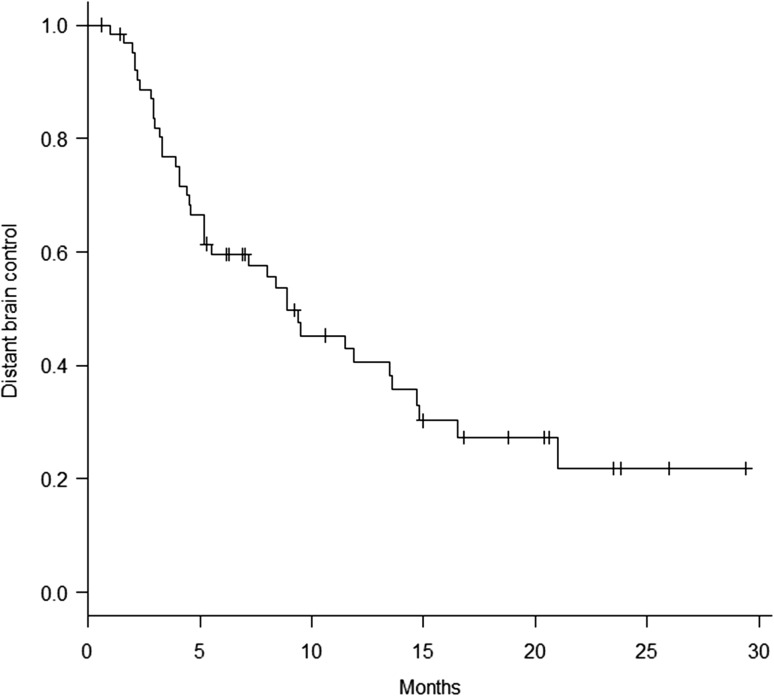
Distant brain control achieved in the 64 assessed patients.

Thirty-nine patients developed distant brain metastases. The median time to distant brain failure was 8.9 months, the distant brain control rate being 59.7% and 40.5% at 6 and 12 months, respectively (Fig. 3). According to univariate and multivariate analyses, being female and having few brain metastases (≤4) were significantly associated with better distant brain control rates (*P* < 0.05).

Salvage treatment was administered to 32 patients, two of whom had local disease only, two both local and distant, and the remaining 28 distant only. Three of the four patients with local recurrence were retreated by surgical resection and the remaining one by HFIGMI–VMAT. Thirty of the 39 patients who developed new lesions were retreated with the following modalities: SRS in 6 cases, repeat HFIGMI–VMAT in 14, SRS and HFIGMI–VMAT in 7, SRS and WBRT in 1, and WBRT in 2. Thus, 27 of the 30 patients (90%) who developed new lesions were retreated with focal therapies (SRS and/or HFIGMI–VMAT).

**Fig. 4.**
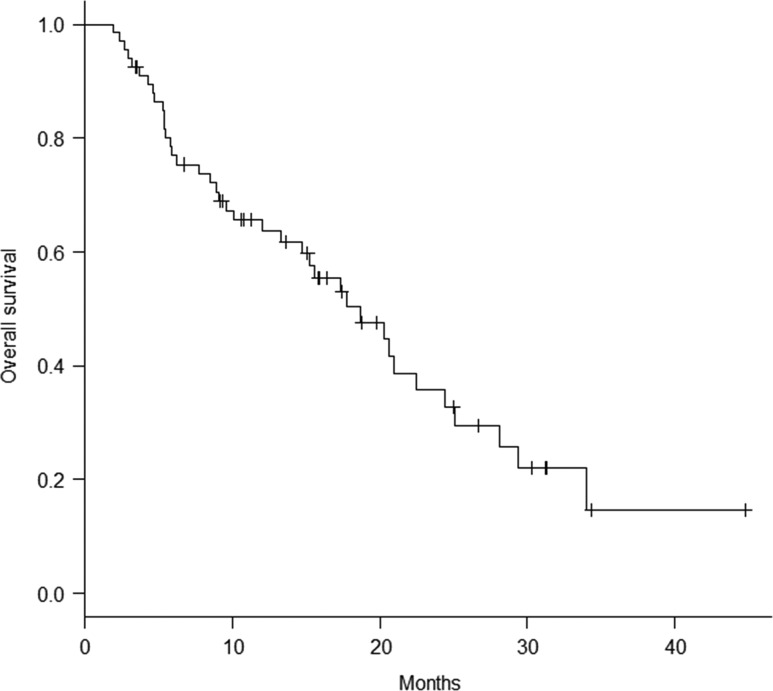
Overall survival of all 67 patients.

Thirty-nine patients were dead and 28 alive at the time of analysis. The median survival time was 18.7 months and the overall survival (OS) rate at 12 months 63.7% (Fig. 4). Eleven patients (28%) died of neurologic causes and 28 (72%) of systemic disease progression. According to univariate and multivariate analyses, being female, few brain metastases (≤4), absence of extracranial metastases, and use of targeted therapy were associated with significantly better OS (*P* < 0.05).

### Toxicity

All patients completed the planned radiation therapy. The median treatment duration was 14 days (range, 10–17 days). Acute toxicities were generally mild (Grade 1–2). One patient developed seizures requiring anticonvulsant therapy (Grade 2), two motor weakness requiring corticosteroid therapy (Grade 2), one dizziness requiring corticosteroid therapy (Grade 2), and four headaches, one of whom required corticosteroid therapy (Grade 2). Overall, acute Grade 2 toxicities occurred in five patients (7.5%). Late radiation necrosis occurred in ten patients (14.9%), three (4.5%) of whom required corticosteroid therapy (Grade 2). No Grade 3 or higher toxicities occurred.

## DISCUSSION

In the present study, we evaluated the treatment outcomes of HFIGMI–VMAT for brain metastases. The treatment procedure and dose fractionation were well tolerated and achieved excellent local control. Being a linear accelerator (linac)-based radiotherapy requiring only non-invasive head masks, HFIGMI–VMAT can be delivered easily in clinics as an alternative to gamma knife (GK)-SRS.

HFIGMI–VMAT has the radiobiological advantage of fractionation, whereas GK-SRS is administered in one to three fractions [25, 26]. Radiosurgical doses ranging from 15 to 24 Gy according to tumor diameters have been widely administered as a single treatment [9, 27]; however, tumor size correlates strongly with local tumor control. Vogelbaum *et al.* reported the following results for GK-SRS treatment using the RTOG 90–05 dosing scheme: 1-year local control rate 85% for 20 mm or smaller (24 Gy), 49% for 21–30 mm (18 Gy), and 45% for 31–40 mm (15 Gy) tumors. Local control rates were significantly lower for tumors >2 cm than for those that were ≤2 cm [28]. Chang *et al.* reported that, after linac-based SRS treatment, the 1-year local control rate was higher for ≤1 cm tumor diameter than for >1 cm (86% vs 56%) [29]. Hypofractionated radiotherapy is employed to maintain local tumor control while minimizing the risk of toxicity to normal tissues [30]; notably, more fractions can be administered with HFIGMI–VMAT than with GK or linac-based SRS. We acknowledge that the applicability of a linear–quadratic (LQ) model for high doses per fraction is controversial [31–33]; however, we used this model to calculate the BED because it is simple and utilitarian. We selected the prescribed dose of 50 Gy to achieve high local control rate for large tumors and assumed that the prescribed dose of 50 Gy in 10 fractions would provide a similar BED to 23 Gy in one fraction SRS or 30 Gy in 10 fractions WBRT combined with 15 Gy in one fraction SRS boost for tumor control, while providing a lower BED for normal tissue toxicity. In this study, we achieved an excellent local control rate at 1 year, especially for ≤2 cm tumors (100%). The median total volume and maximum diameter of brain metastases were 1.0 cm^3^ and 1.2 cm, and the majority of lesions were small, which may have contributed to the high local control rate; however, we achieved high local control rates even in tumors >2 cm (83.3%). Additionally, the number of lesions had no significant influence on local control. This study did not include tumors that were >3 cm and therefore did not assess safety and efficacy of this treatment for such large tumors; however, an additional possible indication for HFIGMI–VMAT is such large tumors. Additional stereotactic approaches (use of non-coplanar arcs and/or smaller PTV margins) may be needed to expand the indications for treating tumors >3 cm; further studies are needed to assess this potential indication.

Because the treatment time of linac-based SRS increases in parallel with the number of brain metastases, most studies of linac-based SRS or stereotactic radiotherapy have reported results in patients with five or fewer brain metastases [30, 34–36]. Use of single isocenter VMAT techniques enables significantly shorter treatment times in patients with multiple brain metastases. In the current study, the treatment time was ~3 min or less of treatment beam-on time in all cases, which is much shorter than for SRS or intensity-modulated radiation therapy treatment [37]. Our results show that the HFIGMI–VMAT technique enables treatment of multiple brain metastases without the limits on number of lesions associated with treatment with linac systems.

The risk of developing new brain metastases increases in parallel with the number of brain metastases present at diagnosis [38, 39]. WBRT has traditionally been the standard treatment for multiple brain metastases; however, the resultant acute toxicities may delay initiation of systemic therapy, and this therapy is associated with an increased risk of late neurocognitive decline [9, 40]. SRS may carry a lower risk of neurocognitive decline; however, whether SRS is indicated in patients with more than four brain metastases is controversial [9]. Several studies have reported the effectiveness of SRS treatment for multiple brain metastases: Yamamoto *et al.* reported the results of a multi-institutional trial in which patients with 1–10 brain metastases were treated with SRS using GK. The OS did not differ between the 2–4 and 5–10 metastases groups [27]. In a retrospective study in which patients with 5–15 brain metastases were treated with GK, Salvetti *et al.* found that the number of lesions did not affect the OS [41]. In the current study, the median number of brain metastases was 5. Although approximately half the patients had developed new brain metastases by 9 months, 30 of the 39 patients who developed new lesions were safely retreated with salvage radiotherapies, and 27 of 30 patients (90%) were retreated with focal therapies (SRS and/or VMAT), enabling deferral of WBRT. The number of lesions was significantly associated with OS in our series; however, the median survival time was 28.1 months in patients with ≤4 metastases and 14.7 months in patients with >4 metastases. These results are favorable compared with those of previous studies of patients with multiple brain metastases treated with WBRT or SRS [7, 37]. The favorable survival may be attributable to recent advances in molecular targeted drugs and improvement in intracranial control with radiation therapy. Several studies have reported that the total tumor volume significantly impacts overall survival [42–44]. The small size of the tumors in the current cohort of patients (median of 1.0 cm^3^ for five metastases) may also have contributed to the favorable survival outcomes.

Acute toxicities of the treatment were generally mild (Grade 1–2), and all patients completed the planned radiation therapy. Delayed radiation necrosis was identified in 10 patients (14.9%), 3 of whom (4.5%) were symptomatic (Grade 2). No Grade 3 or higher toxicities occurred; the incidence of radiation necrosis was similar to that reported for SRS [6]. In addition, this treatment was well tolerated by all 9 patients who had previously received WBRT, suggesting the potential role of this treatment as salvage therapy after prior WBRT. In this study, we selected a 3 mm PTV margin to allow for positional deviation arising from set-up and mechanical and imaging errors in the setting of a single isocenter VMAT approach for multiple lesions; however, most previous studies of SRS have used smaller margins (0–2 mm). Kirkpatrick *et al.* reported that radiation necrosis occurred more frequently in the 3 mm than in the 1 mm PTV margin group, whereas local recurrence rate did not differ significantly between these groups [45]. Use of a smaller PTV margin may further decrease the incidence of radiation necrosis associated with HFIGMI–VMAT treatment. Nichol *et al.* reported the results of a multi-institutional trial in which patients with 1–10 brain metastases were treated with volumetric radiosurgery concurrently with WBRT. The incidence of severe radiation necrosis (Grade 3–5) was 10% [14]. Although the eligibility criteria (diameter ≤3 cm and total tumor volume ≤15 cm^3^) and prescribed dose (47.5 Gy in five fractions to metastases) were similar to those of our study, the number of fractions differed, and resultant differences in fraction size and flatness of dose delivery likely affected the incidence of radiation necrosis. A longer treatment duration is a disadvantage of our treatment schedule; however, 10 fractions are safer and more feasible for the prescribed dose of 50 Gy to minimize toxicity.

Neurocognitive decline is one of the most concerning late toxicities of cranial radiation. Chang *et al.* reported the addition of WBRT to SRS significantly increases neurocognitive decline [46]. In contrast, Aoyama *et al.* demonstrated that progression of brain tumor has a greater impact on neurocognitive decline than WBRT [47]. In the present study, half the patients had developed new brain metastases by 9 months, most of whom were retreated safely with focal therapies without WBRT. We did not assess neurocognitive function and therefore could not draw any conclusions about preservation of neurocognitive function with HFIGMI–VMAT treatment; further studies focusing on neurocognitive function are needed.

The limitations of this study are that it was a small single-institution retrospective analysis with heterogeneous participants. However, our results show that HFIGMI–VMAT has the potential to treat multiple and large brain metastases. Further prospective studies are needed to confirm these findings.

In conclusion, HFIGMI–VMAT with flat dose distribution and a relatively larger PTV margin has the advantages of patient comfort, fractionated treatment regimens, and short treatment time, even in patients with multiple brain metastases. It achieves an excellent rate of local control and the toxicities are tolerable. Thus, this technique is a promising treatment alternative to GK-SRS in patients with multiple and large brain metastases attending clinics with linac systems.
